# Draft genome sequence data and analysis of *Shinella* sp. strain JR1-6 isolated from nitrate- and radionuclide-contaminated groundwater in Russia

**DOI:** 10.1016/j.dib.2019.104319

**Published:** 2019-07-25

**Authors:** Denis S. Grouzdev, Tamara L. Babich, Diyana S. Sokolova, Tatiyana P. Tourova, Andrey B. Poltaraus, Tamara N. Nazina

**Affiliations:** a Institute of Bioengineering, Research Center of Biotechnology, Russian Academy of Sciences, Moscow, Russian Federation; b Winogradsky Institute of Microbiology, Research Center of Biotechnology, Russian Academy of Sciences, Moscow, Russian Federation; c Engelhardt Institute of Molecular Biology, Russian Academy of Sciences, Moscow, Russian Federation; d V.I. Vernadsky Institute of Geochemistry and Analytical Chemistry of Russian Academу of Sciences, Moscow, Russian Federation

**Keywords:** Draft genome, *Shinella* sp., Groundwater, Denitrification, Metal resistance

## Abstract

*Shinella* sp. strain JR1-6 is a Gram-negative, facultatively anaerobic, non-spore-forming, motile, rod-shaped bacterium isolated from radionuclide- and nitrate-contaminated groundwater. This bacterium reduces nitrate to N2. Strain JR1-6 has potential for removal of nitrate contamination, which is the main reason for the interest in sequencing its genome. Here, we present a set of features of *Shinella* sp. strain JR1-6, together with the description of its genomic sequencing and annotation. The draft genome of strain JR1-6 has a size of ∼7.09 Mb and contains 6,945 genes, including 62 RNA genes. In the genome of strain JR1-6, the genes were revealed encoding nitrate reduction to N2, as well as the genes associated with metal resistance, showing its adaptation to the conditions of the environment and possible role in nitrate removal from contaminated groundwater. The draft genome sequence of *Shinella* sp. strain JR1-6 is available at DDBJ/EMBL/GenBank under the accession no. SHMI00000000.

**Table:** Specifications Table

Subject area	Biology
More specific subject area	Microbiology and genomics.
Type of data	Genome sequencing data, table, image and figure.
How data was acquired	Genome sequencing: HiSeq 1500 platform (Illumina). Bioinformatics approaches: NCBI Prokaryotic Genomes Automatic Annotation Pipeline (PGAAP) and the Rapid Annotation using Subsystems Technology (RAST) server. Transmission electron microscopy: JEOL JEM-1010, Japan.
Data format	Raw, analyzed and deposited.
Experimental factors	A new strain was isolated, cultured, observed under transmission electron microscope. Genome was sequenced and annotated.
Experimental features	Draft genome sequencing was performed according to Illumina sequencing protocols for DNA-seq followed by annotation and gene description.
Sample source location	The strain was isolated from a groundwater sample at 55°38′ N, 60°47′ E, Ozyorsk town, South Urals, Russia.
Data accessibility	The draft genome sequence of *Shinella* sp. strain JR1-6 is available at DDBJ/EMBL/GenBank under the accession no. SHMI00000000. The version described in this paper is version SHMI01000000. The raw FASTQ reads have been deposited in the NCBI SRA database under the accession no. SRR9587904.

**Table:** 

**Value of the data** • The data obtained might increase the molecular information on bacteria inhabiting groundwater highly contaminated with nitrate and radionuclides. • The draft genome sequence of *Shinella* sp. strain JR1-6 will provide insights into the genetic determinants involved in heavy metal and antibiotic resistance in bacteria of the genus *Shinella*. • Data of genome sequencing of *Shinella* sp. JR1-6 can be used for further understanding of the genomic potential of the strain and elucidation of its possible biotechnological application for nitrate removal from contaminated water.

## 1 Data

**Fig. 1:**
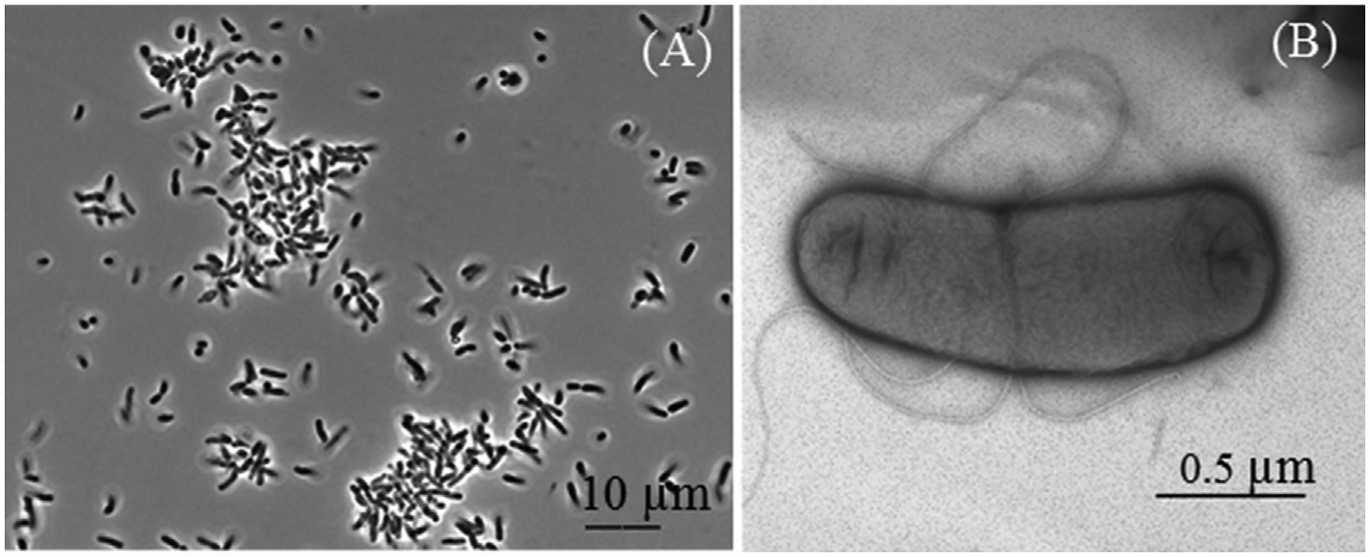
Phase contrast photomicrograph of the cells of *Shinella* sp. strain JR1-6 (A) grown aerobically at 28 °C in TEG medium for 72 h and a micrograph (B) of flagellated cells contrasted with 1% phosphotungstic acid obtained by transmission electron microscopy on JEM-100C.

**Fig. 2:**
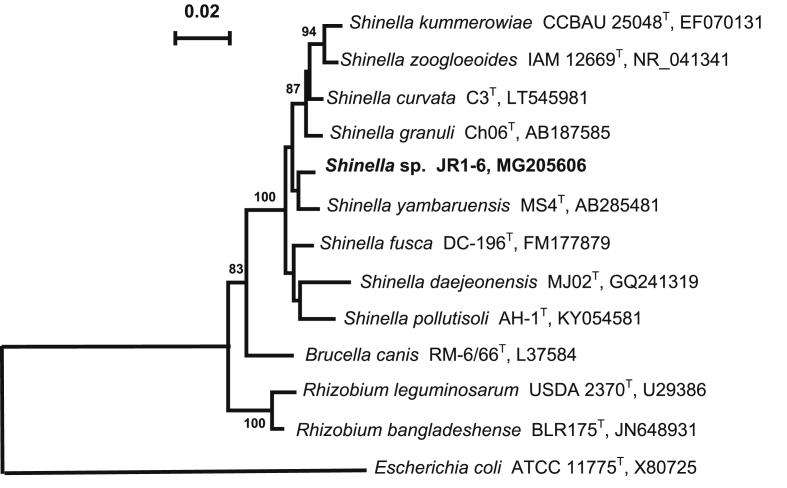
Neighbour-joining tree based on the 16S rRNA gene sequences, showing the phylogenetic position of strain JR1-6 and related members of the genus *Shinella* and genera of the family *Rhizobiaceae*. Bootstrap values are based on 1000 replicates; values > 50% are shown. Bar, 0.02 substitutions per nucleotide position.

**Fig. 3:**
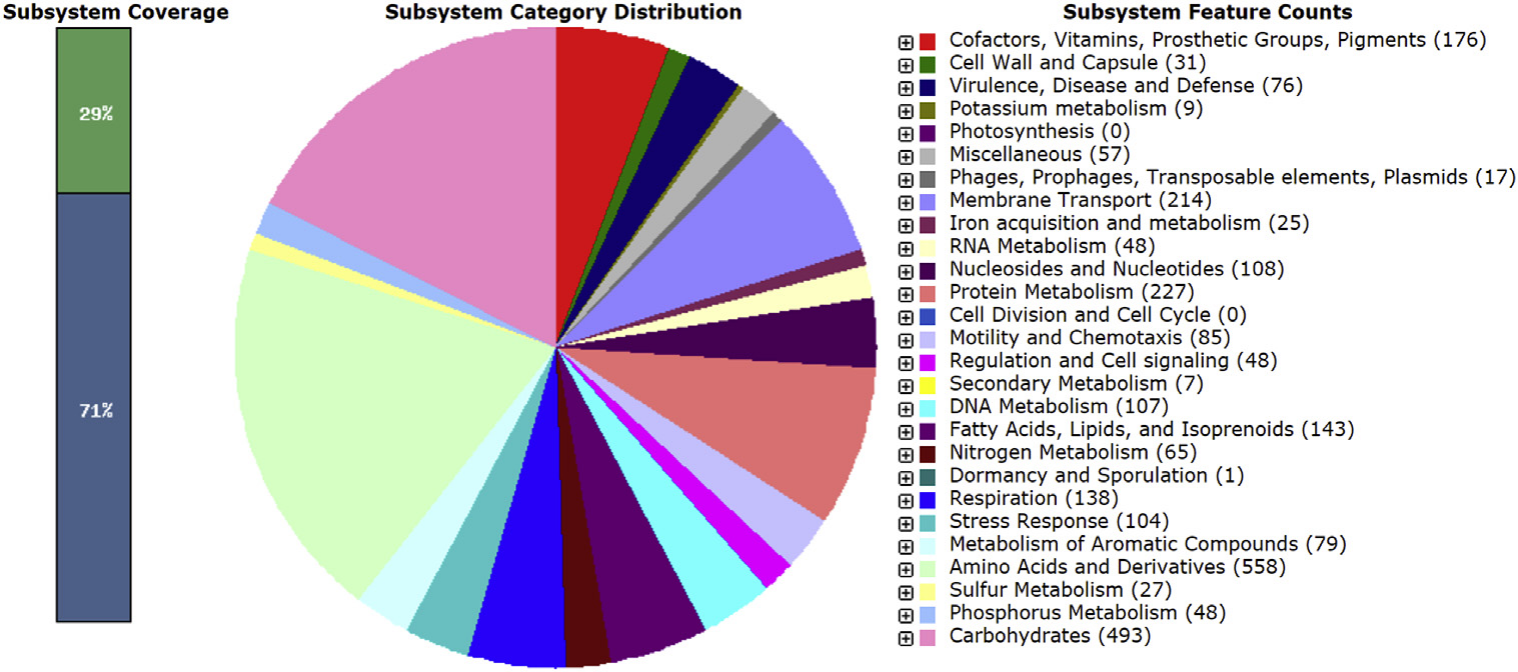
Subsystems of *Shinella* sp. JR1-6 based on SEED database.

In the present work, we report the draft genome sequence data and genome annotation of a denitrifying bacterial strain JR1-6 (=VKM B-3307) isolated from a groundwater sample collected near the surface reservoir for liquid radioactive waste (Ozyorsk, South Urals, Russia) (55°38′ N 60°47′ E) [1]. Strain JR1-6 was chosen for genome sequencing in order to identify the genetic determinants providing for its occurrence in the environment contaminated with nitrate and radionuclides and to elucidate its possible application in wastewater treatment biotechnologies for nitrate and nitrous oxide removal. The cells of the strain JR1-6 grown in liquid TEG medium with bacto-trypton, yeast extract, and glucose were non-spore-forming rods 0.65–0.96 × 1.3–3.5 μm, motile at the early stage of incubation (Fig. 1). The strain grew optimally at 23–28 °C, pH 7–8, and 1–1.5% NaCl (Table 1). In the medium with acetate and nitrate the strain reduced nitrate to nitrite and then to dinitrogen gas. Strain JR1-6 was a member of the genus *Shinella* within the family *Rhizobiaceae* of the class *Alphaproteobacteria* (Table 1) [2], [3], [4], [5], [6], [7]. Its 16S rRNA gene sequence (GenBank accession number MG205606) showed the highest similarity with respective sequence of *Shinella yambaruensis* MS4^T^ (98.8%) (Fig. 2). The genus *Shinella* contains eight species: *S. granuli, S. zoogloeoides, S. kummerowiae*, *S. yambaruensis*, *S. fusca*, *S. daejeonensis, S. curvata,* and *S. pollutisoli* [7], [8], [9], [10]. Members of this genus are aerobic organotrophs, which have been isolated from an anaerobic sludge blanket reactor and a sewage treatment system, from domestic waste compost, root nodules, and from polluted soil. Nitrate is reduced and supports anaerobic growth of *S. fusca* and *S. daejeonensis.* Since the genome of the *S. yambaruensis* type strain is not represented in the NCBI database, unequivocal determination of the species position of the new strain JR1-6 was impossible. The features for the draft genome sequence of *Shinella* sp. JR1-6 are summarized in Table 2**.** The draft genome sequence of *Shinella* sp. strain JR1-6 contained 6,945 genes, of which 6,701 were protein-coding sequences, 182 were pseudo genes, and 58 coded RNAs (tRNAs, 5S, 16S, and 23S) and 4 ncRNAs. Most of the annotated genes determined the synthesis of amino acids and derivatives (558), carbohydrate metabolism (493), protein metabolism (227), membrane transport (214), and synthesis of cofactors, vitamins, prosthetic groups and pigments (176) (Fig. 3). The genome of *Shinella* sp. JR1-6 contained at least 7 plasmids, since 7 different *repABC* gene clusters located on 7 different contigs were detected. In the genome of *Shinella* sp. strain JR1-6 the genes were revealed encoding nitrate reduction to N_2_, as well as the genes responsible for utilization of various monosaccharides and proteins. Several genes responsible for cobalt, zinc, cadmium, and mercury resistance were also observed. Phenotypic and genomic data set of *Shinella* sp. strain JR1-6 indicates its adaptation to the conditions of the environment and its possible role in nitrate removal from contaminated groundwater. The Whole Genome Shotgun project of *Shinella* sp. JR1-6 has been deposited at DDBJ/EMBL/GenBank under the accession no. SHMI00000000 and the release date of its GenBank Data is February 26, 2019. The raw FASTQ reads have been deposited in the NCBI SRA database under the accession no. SRR9587904.

**Table 1:** Classification and general features of *Shinella* sp. strain JR1-6 according to the MIGS recommendations [2].

MIGS ID	Property	Term	Evidence code^a^
	Classification	Domain *Bacteria*	TAS [3]
		Phylum *Proteobacteria*	TAS [4]
		Class *Alphaproteobacteria*	TAS [5]
		Order *Rhizobiales*	TAS [6]
		Family *Rhizobiaceae*	TAS [6]
		Genus *Shinella*	TAS [7]
		Species *Shinella* sp.	IDA
		Strain: JR1-6 (VKM В-3222)	TAS
	Gram stain	Gram-negative	IDA
	Cell shape	Rod	IDA
	Motility	Motile	IDA
	Sporulation	Non-spore-forming	IDA
	Temperature range	16–37 °C	IDA
	Optimum temperature	23–28 °C	IDA
	pH range; optimum	6–9; 7–7.5	IDA
	Carbon source	D-arabinose, D-cellobiose, D-glucose, D-fructose, D-ribose, D-sucrose, D-trehalose, D-xylose, L-valine, leucine	IDA
	Energy source	Chemoheterotrophic	IDA
MIGS-6	Habitat	Groundwater	IDA
MIGS-6.3	Salinity; optimum	Up to 5% NaCl, 1–1.5% NaCl (w/v)	IDA
MIGS-22	Oxygen requirement	Aerobic, facultatively anaerobic	IDA
MIGS-15	Biotic relationship	Free-living	IDA
MIGS-14	Pathogenicity	None	NAS
MIGS-4	Geographic location	Russia/South Urals/Ozyorsk town	IDA
MIGS-5	Sample collection	2011	IDA
MIGS-4.1	Latitude	55°38′ N	IDA
MIGS-4.2	Longitude	60°47′ E	IDA
MIGS-4.4	Depth	44 m	IDA

a Evidence codes - IDA: Inferred from Direct Assay; TAS: Traceable Author Statement (i.e., a direct report exists in the literature); NAS: Non-traceable Author Statement (i.e., not directly observed for the living, isolated sample, but based on a generally accepted property for the species, or anecdotal evidence). These evidence codes are from the Gene Ontology project (cite this reference).

**Table 2:** Genome statistics of *Shinella* sp. strain JR1-6.

Attribute	*Shinella* sp. JR1-6	
	Value	% of Total
Genome size (Mb)	7093386	100.00
DNA coding (bp)	6339649	89.37
DNA G + C (bp)	4509941	63.58
DNA scaffolds	131	100.00
Total genes	6945	100.00
Protein-coding genes	6701	96.49
RNA genes	62	0.89
Pseudo genes	182	2.62
Genes in internal clusters	–	–
Genes with function prediction	5671	81.65
Genes assigned to COGs	1255	18.07
Genes with Pfam domains	5682	81.81
Genes with signal peptides	888	12.89
Genes with transmembrane helices	1519	21.87
CRISPR repeats	73	–

## 2 Experimental design, materials, and methods

### 2.1 Isolation of the strain JR1-6

Strain JR1-6 was isolated from a groundwater sample contaminated with nitrate, sulfate, acetate, and radionuclides. At the time of sampling, pH and Eh of the groundwater were 7.9 and + 200 mV, respectively. The sample was collected at the observation well 1/69 from the depth of 44 m at a distance 3.2 km from the Karachai Lake (Ozyorsk town, South Urals, Russia) [1]. The strain was purified by successive transfers from the liquid TEG medium containing bacto-trypton (5.0 g L^−1^), yeast extract (1.0 g L^−1^), glucose (5.0 g L^−1^), and distilled water (1 L, pH 7.0) to solid TEG medium with agar-agar (15.0 g L^−1^). Bacteria were incubated at 22–28 °C. Strain JR1-6 was deposited in the All-Russian Collection of Microorganisms as VKM В-3307.

### 2.2 DNA isolation and sequencing

Biomass of the strain JR1-6 was grown in TEG liquid medium for 72 h at 28 °C. The cells were harvested by centrifugation. Integrity of the cells was accessed by transmission electron microscopy (JEOL JEM-1010, Japan) of bacteria negatively stained with 1% phosphotungstic acid (Fig. 1B). Genomic DNA was extracted according to the method of Wilson [11], with minor modifications. The cell pellet was resuspended in 400 μl of TE-buffer. Thereafter, 25 μL of 10% SDS and 20 μL of proteinase K solution were added and the mixture was incubated at 37 °C for 60 min. After incubation, 125 μL of 4 M NaCl, 160 μL of 5% CTAB and 20 μL of RNase (10 mg/mL) were added. The mixture was then incubated for 10 min at 65 °C and cooled to room temperature; thereafter, the mixture was treated with chloroform followed by centrifugation for 10 min at 9000 × *g*. DNA was extracted from the supernatant by adding 0.6 volume of isopropanol. The dried DNA sample was dissolved in 50 μL of MQ. The libraries were constructed with the NEBNext DNA library prep reagent set for Illumina, according to the protocol for the kit. Next-generation shotgun-sequencing of the genomic DNA was carried out using the Illumina HiSeq 1500 platform (Illumina Inc., USA) with 250-bp single-end reads.

### 2.3 Genome assembly and annotation

A total of 1,734,433 reads were obtained from JR1-6. Raw sequence reads were quality-checked with FastQC v.11.7 (https://www.bioinformatics.babraham.ac.uk/projects/fastqc/), and low-quality reads were trimmed using Trimmomatic v. 0.36 [12]. Subsequently, the quality-filtered reads were *de novo* assembled with SPAdes version 3.11.0 using the default settings [13]. The final assembled 7,093,386-bp-long genome comprised of 131 scaffolds, with an N_50_ value of 237,993  bp and an average coverage of 41 × . Identification of protein-coding sequences and primary annotation was performed using the NCBI Prokaryotic Genome Automatic Annotation Pipeline (PGAAP) [14]. Additional gene prediction and functional annotation were performed in the Rapid Annotation using Subsystems Technology (RAST) server [15].

## Acknowledgements

Genome sequencing of the strain was supported by the Russian Science Foundation (grant 17-17-01212). Physiological and taxonomic studies of the strain were supported by the Ministry of Science and Higher Education of the Russian Federation. The funds had no role in the study design, data collection and interpretation, or the decision to submit the work for publication.

## Conflict of interest

The authors declare that they have no known competing financial interests or personal relationships that could have appeared to influence the work reported in this paper.
